# Plasma Proteomic Patterns Show Sex Differences in Early Concentric Left Ventricular Remodeling

**DOI:** 10.1161/CIRCHEARTFAILURE.122.010255

**Published:** 2023-06-29

**Authors:** Anne-Mar van Ommen, Ernest Diez Benavente, N. Charlotte Onland-Moret, Gideon B. Valstar, Maarten J. Cramer, Frans H. Rutten, Arco J. Teske, Roxana Menken, Leonard Hofstra, Igor I. Tulevski, Nancy Sweitzer, G. Aernout Somsen, Hester M. den Ruijter

**Affiliations:** Laboratory of Experimental Cardiology (A.-M.v.O., E.D.B., G.B.V., H.M.d.R.), University Medical Center Utrecht, Utrecht University, the Netherlands.; Department of Epidemiology, Julius Center for Health Sciences and Primary Care (N.C.O.-M., F.H.R.), University Medical Center Utrecht, Utrecht University, the Netherlands.; Clinical Cardiology Department (M.J.C., A.J.T.), University Medical Center Utrecht, Utrecht University, the Netherlands.; Cardiology Centers of the Netherlands (R.M., L.H., I.I.T., G.A.S.).; Department of Medicine, Washington University School of Medicine, St. Louis, MO (N.S.).

**Keywords:** echocardiography, heart failure, interferons, prevention & control, prognosis, proteomics, sex characteristics

## Abstract

Concentric remodeling (cRM) can precede heart failure with preserved ejection fraction (HFpEF), a condition prevalent in women.

**METHODS:**

Patients (n=60 593, 54.2% women) visiting outpatient clinics of Cardiology Centers of the Netherlands were analyzed for cRM, HFpEF development, and mortality risk. We studied risk factors for relative wall thickness both sex-stratified and in women and men combined. Biomarker profiling was performed (4534 plasma proteins) in a substudy involving 557 patients (65.4% women) to identify pathways involved in cRM.

**RESULTS:**

cRM was present in 23.5% of women and 27.6% of men and associated with developing HFpEF (HR, 2.15 [95% CI, 1.51–2.99]) and mortality risk (HR, 1.09 [95% CI, 1.00–1.19]) in both sexes. Age, heart rate, and hypertension were statistically significantly stronger risk factors for relative wall thickness in women than men. Higher circulating levels of IFNA5 (interferon alpha-5) were associated with higher relative wall thickness in women only. Pathway analysis revealed differential pathway activation by sex and increased expression of inflammatory pathways in women.

**CONCLUSIONS:**

cRM is prevalent in approximately 1 in 4 women and men visiting outpatient cardiology clinics and associated with HFpEF development and mortality risk in both sexes. Known risk factors for cRM were more strongly associated in women than men. Proteomic analysis revealed inflammatory pathway activation in women, with a central role for IFNA5. Differential biologic pathway activation by sex in cRM may contribute to the female predominance of HFpEF and holds promise for identification of new therapeutic avenues for prevention and treatment of HFpEF.

**REGISTRATION:**

URL: https://www.clinicaltrials.gov; Unique identifier: NCT001747.

**WHAT IS NEW?**

- Concentric remodeling of the heart is common during ageing and is a feature that can precede heart failure with preserved ejection fraction. We show that concentric remodeling is equally prevalent among men and women visiting outpatient clinics and predicts incident heart failure and mortality in both. Risk factor relations with concentric remodeling were stronger in women and plasma proteomics revealed strong inflammatory pathway activation in women with concentric remodeling, with a central role for IFNA5 (interferon alpha-5). This highlights the importance of sex stratification in biomarker studies for prevention of heart failure.

**WHAT ARE THE CLINICAL IMPLICATIONS?**

- There are promising studies targeting inflammation to treat or prevent cardiovascular diseases. Slowing down inflammation may limit progression of concentric remodeling towards heart failure with preserved ejection fraction in women. A biomarker such as IFNA5 could be used to identify women at high risk for heart failure as these individuals would be most likely to benefit from anti-inflammatory therapies.

Women are twice as likely to have heart failure with preserved ejection fraction (HFpEF) than men,^1^ whereas men are more often diagnosed with heart failure with reduced ejection fraction (HFrEF). Both heart failure types have a poor prognosis, with comparable mortality rates.^2,3^ Decades of research on HFpEF has resulted in only a few therapies which improve prognosis, while multiple therapeutic options are available in HFrEF.^4^ Therefore, HFpEF is a significant unmet need in cardiovascular medicine. Public health implications are significant, as prevalence is rising. The different heart failure (HF) profiles in women and men^1^ might be explained by sex-related changes in the biology of ventricular geometry during aging.^5,6^

The heart changes geometrically in both aging and HF development. Concentric remodeling (cRM) and concentric left ventricular hypertrophy (cLVH), both marked by an increased relative wall thickness (RWT), are frequently found in HFpEF. The prevalence of cRM is ranging from 14% to 28% in HFpEF populations.^7^ cLVH is associated with worse outcome in HFpEF, but cRM is not.^5,6^ However, cRM is more prevalent than cLVH in the general population,^8^ and especially in high-risk populations the prognostic implications of cRM are unclear. Cellular hypertrophy, increased extracellular matrix, and fibrosis can all drive structural remodeling, and are in turn caused by pressure overload, systemic inflammation, and endothelial dysfunction.^9^ We know that women more often develop cRM and cLVH in response to pressure overload than men^10,11^; coronary microvascular dysfunction is also more common in women. Investigating processes ongoing in women and men with cRM may clarify the biology of early disease in high-risk individuals. The use of unselected high-throughput plasma proteomic assays may reveal early reversible processes not previously identified, potentially preceding fibrosis and microvascular dysfunction. Furthermore, it is important that sex-specific information on biomarkers at a disease stage where prevention from progression to overt disease is still feasible becomes available.

We studied to what extent a cRM phenotype increases HFpEF and mortality risk in a large high-risk cohort with adequate numbers of women and men. In addition, we identified clinical risk factors of cRM. Finally, we studied the plasma proteome in a subset of patients, to examine proteins associated with early structural remodeling in those at risk for HFpEF (visual overview Figure S1).

## METHODS

### Study Population

Longitudinal data from patients (n=109 151) visiting 13 outpatient clinics of Cardiology Centers of the Netherlands (CCN) between 2007 and 2018 were extracted. A full description of the CCN clinical health record dataset, which was retrieved under implied consent, and in accordance with the Dutch Personal Data Protection Act, can be found elsewhere.^12^ Patients were referred by their general practitioner for cardiac work-up including electrocardiography, exercise testing, and echocardiography, followed by consultation with a cardiologist. We excluded patients without available echocardiography/RWT, patients younger than 45 years, and patients already diagnosed with HF, leaving 60 593 patients (54.2% women) for analyses (Figure S2A).

Additionally, between 2016 and 2019, in a subsample of patients (n=880, 68.6% women) who visited CCN at the Utrecht location, blood was drawn for a biomarker study (Dutch Trial Register number 21717; Figure S2B). These patients underwent the same work-up, but participants with average E/e' ratio ≥8 were oversampled, as described previously.^13^ This study was approved by the local medical ethics committee (16-290/M) and conducted according to the declaration of Helsinki.

The data that support the findings of this study are available from the corresponding author upon reasonable request.

### Assessment of RWT and Remodeling Patterns

As part of the clinical assessment, comprehensive transthoracic echocardiography (Vivid E6 or E7, General Electric Medical Systems, Horten, Norway) was performed by trained sonographers, and interpreted by the treating cardiologist.^14^ Measurements included parasternal long axis M-mode diameters of septal and posterior wall (left ventricular posterior wall dimension) and left ventricle diameter at end diastole. Body surface area was calculated,^15^ and used to index left ventricular mass (LVMI).^16^ Left ventricular hypertrophy (LVH) was defined as an LVMI >95 gram/m^2^ in women, and >115 gram/m^2^ in men.^14^ We calculated relative wall thickness (RWT) as percentage with the formula ([2×left ventricular posterior wall dimension]/left ventricle diameter at end diastole)×100. We classified patients into 4 different geometry patterns: (1) cRM=RWT>42%, no LVH; (2) cLVH=RWT>42% and LVH; (3) eccentric LVH=RWT≤42% and LVH; and (4) normal geometry=RWT≤42%, no LVH.

### Outcome Assessment of Heart Failure and Survival

Enrolled participants with more than 1 visit to CCN were analyzed for subsequent HF outcomes. We defined HF as having a diagnosis of HF registered by the treating cardiologist. HFpEF and HFrEF were classified based on echocardiography derived LVEF ≥50% and <50% within 1 year of diagnosis, respectively, as previously described.^4,17^ Types of HF included HFpEF, HFrEF, and the ones that had HF without LVEF available. Patients without HF were censored at the last available visit (up to March 1, 2018).

Follow-up for all-cause mortality was performed up to February 11, 2021 through linkage with the national death registry. Follow-up for patients who were alive was censored at this date.

### Traditional Cardiovascular Risk Factors

Potential risk factors for cRM were obtained from the CCN electronic health records. Rate-pressure product at rest, exercise, and the delta between exercise and rest rate-pressure product was derived from the exercise test, which was performed in >70% of patients. Antihypertensive medication was defined as the use of an ACE-inhibitor, angiotensin II receptor blocker, thiazide diuretic, spironolactone or calcium channel blocker, or a combination.

### Proteomics

Plasma (ethylenediamine tetraacetic acid) samples of 606 participants were sent (frozen and on dry ice with temperature monitoring) to SomaLogic (Boulder, Colorado) for SomaScan V4 assay measurement, a platform for quantifying 5284 reagents, as described previously.^18^

Raw data from SomaScan was first normalized to remove hybridization variation within a run. This was followed by median normalization across calibrated samples to remove other assay biases within the run. Overall scaling was then performed on a per-plate basis to remove overall intensity differences between runs followed by calibration to correct for assay differences between runs. Finally, median normalization to a reference was performed on the quality control, buffer, and individual samples as per SomaLogic protocol.

Data were log-transformed and center-scaled by dividing the protein average measurement by the SD according to instructions in the pipeline (https://github.com/SomaLogic/SomaDataIO). A total of 5284 SOMAmers were measured in 606 samples, 305 SOMAmers were excluded as they did not represent human proteins. Furthermore, 445 human proteins were excluded according to the quality control ratio (0.8–1.2). A total of 47 samples were excluded due to missing RWT data and 2 outlier samples were excluded based on normalization criteria (0.4–2.5) as per SomaScan requirements. In total, 4534 proteins in 557 participants were available for analysis (Figure S3).

### Statistical Analysis

Continuous variables are reported as mean with SD, or median and interquartile range, depending on normality. Categorical variables are expressed as counts and percentages. All datasets were multiply imputed using the mice package to prevent selection bias due to missing data,^19^ except for the proteomics dataset. The amount of missing data was limited, and never exceeded 50%. Average missingness was 6.7% in the proteomics subset and 8.1% in the CCN dataset (Table S1).

Cox proportional hazards models were used to assess the relation between cRM, cLVH and eccentric LVH, and the risk of HF, HFpEF, HFrEF, and mortality risk in women and men separately, with the normal geometry category as the reference group. In addition, we adjusted for potential confounders: age, systolic blood pressure, body mass index, diabetes, dyslipidemia, smoking, hypertension, and estimated glomerular filtration rate. We tested whether the models for women and men differed statistically, by adding an interaction term of the determinant and each covariable with sex to a fully adjusted model including both women and men and compared models using the Wald test.

To identify risk factors associated with cRM, we used sex-stratified linear regression models with RWT as outcome, excluding 5892 patients with LVH. Continuous variables were analyzed per SD increase. Multivariable adjustment for confounders was performed as reported in the table legends. Sex-interaction testing was performed as described above. To assess effects of LVH on the associations, we repeated the risk factor analysis in the full cohort.

For the proteomics analyses, we first performed sex-stratified univariable linear regression with RWT as outcome and proteins as determinants, excluding 37 persons with LVH. We then corrected the models for age. Next, we repeated the analyses including persons with LVH. We calculated a standard *P* value for each model and additionally calculated a Benjamin-Hochberg adjusted *P* value to correct for multiple testing. Sex-interaction was tested as described above. Using proteins associated with RWT based on significant standard *P* values in the age-corrected sex-stratified linear regression models, excluding the participants with LVH, we then performed pathway analyses using *ClusterProfiler* package in R.^20^ We assessed pathways significantly associated with cRM, which we quantified using −log 10 p values.

We performed all analyses in R (version 4.0.3). A *P* value of <0.05 was considered statistically significant.

## RESULTS

### Demographics of Concentric Remodeling

CCN patients included in this analysis (n=60 593, 54.2% women) had a mean age of 61 years (±SD 10). cRM was common and present in 7718 women (23.5%), and 7655 men (27.6%). cLVH was relatively rare (5.2% in women and 3.9% in men; Table 1). Women and men with cRM were on average older, had higher systolic blood pressure, were more often diagnosed with hypertension and diabetes, and were more often prescribed statins, B-blockers, and antihypertensive medications than those with normal geometry (Table 1). Women and men with cLVH had the highest systolic blood pressure (157 mm Hg) and the highest prevalence of hypertension, compared with all other morphological groups. Although the proportion of women with hypertension was consistently 2% to 4% higher compared with men (in all groups), they less frequently (1% to 8%) received antihypertensive medication. Women in all groups received statin therapy less often than men (statins prescribed in 26.7%–46.1% of women and 38.2%–53.8% of men). Women in all groups had higher total cholesterol levels compared with men (Table 1).

**Table 1.**
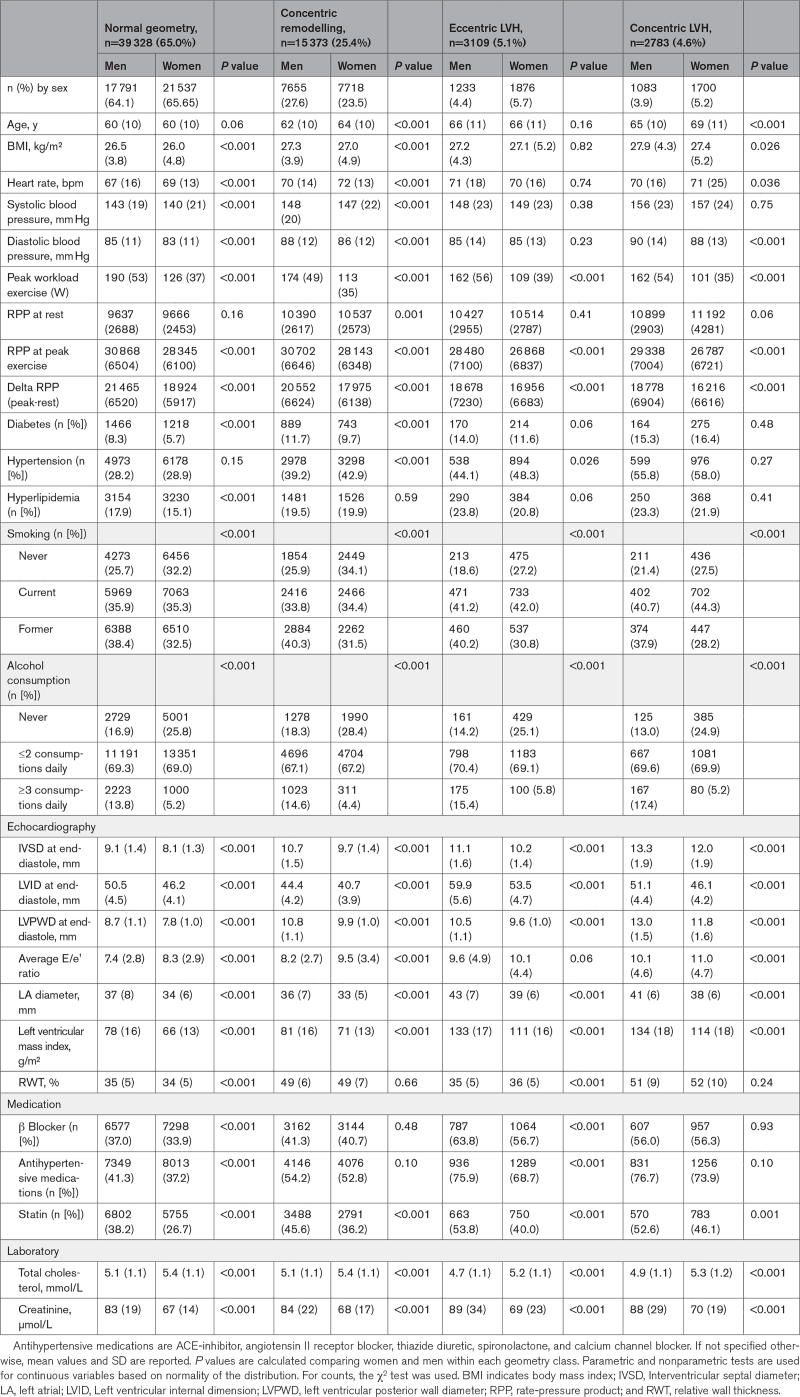
Baseline Characteristics of 60 593 Women and Men Visiting the Cardiology Centers of the Netherlands Dataset Stratified by Sex and Ventricular Geometry Class

### Incident HF

A total of 24 624 (40.6%) patients had a follow-up visit. After a median follow-up of 19 months (interquartile range: 4–53 months), there were 704 HF cases. Of these, 312 cases were HFpEF (54.5% women) and 137 HFrEF (27.1% women). Adjusted overall HF risk was not increased by having cRM at baseline when combining women and men (HR, 1.27 [95% CI, 0.91–1.77]); however, there was significant sex-interaction (*P*_sex-interaction_=0.034). Splitting the results for women and men revealed an increased overall HF risk for cRM in women only (HR, 1.72 [95% CI, 1.23–2.40]). cRM also increased the risk of incident HFpEF for women and men combined (HR, 1.40 [95% CI, 1.00–1.98]), but there was no significant sex-interaction (*P*_sex-interaction_=0.20). Eccentric LVH and cLVH were both significantly associated with incident HF and HFpEF, in both combined and sex-stratified analyses. We found slightly higher risks for these patterns in men than women (Table 2). Unadjusted results and associations of geometry patterns with HFrEF are in Tables S2 and S3.

**Table 2.**
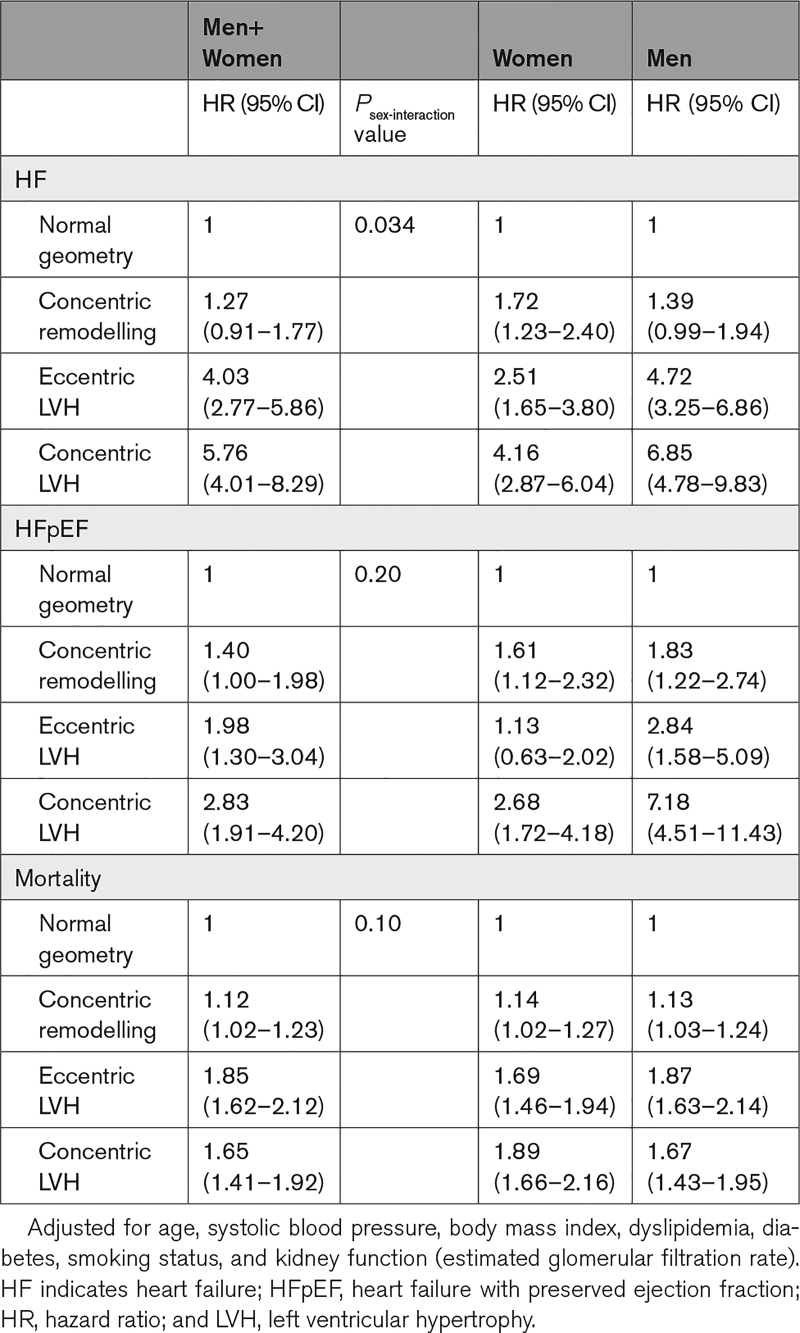
Cox Regression Analyses of Geometry Patterns With Heart Failure, HFpEF, and Mortality

### All-Cause Mortality

Statistics Netherlands successfully linked 96.1% (n=58 239) of the study population. A total of 4324 persons (7.4%) died during 6 years (interquartile range, 4–8 years) follow-up (46.4% women). Adjusted mortality risk was increased by having cRM at baseline when combining women and men (HR, 1.12 [95% CI, 1.02–1.23]), without significant sex-interaction (*P*_sex-interaction_=0.10). Eccentric LVH, and next cLVH, showed a more severe increased mortality risk than cRM (HR, 1.85 [95% CI, 1.62–2.12], and HR, 1.65 [95% CI, 1.41–1.92]; Table 2). Unadjusted results are in Table S2.

### Clinical Risk Factors for Higher RWT

Advancing age, higher body mass index, elevated resting heart rate, systolic- and diastolic blood pressure, and prevalent diabetes and hypertension, as well as prescription of statins, B-blockers, and antihypertensive medications were associated with higher RWT after multivariable adjustment (Table 3). The association of age with higher RWT (per point % increase) was stronger in women (β women=2.16 [95% CI, 2.07–2.25] than men [β men=1.16 [95% CI, 1.06–1.26] per SD increase in age, *P*_sex-interaction_≤0.001). Systolic and diastolic blood pressures were stronger risk factors in men than in women, while higher heart rate, hypertension, prescription of statins, B-blockers, and antihypertensive medications were significantly stronger associated with higher RWT in women (Tables 3 and 4). When we used cRM as binary outcome, the associations of systolic blood pressure and DBP with having cRM were also statistically stronger in men than women, but for hypertension there was no sex-interaction (Table S4). Alcohol consumption in women (β women=−0.96 [95% CI, −1.45 to −0.48] for ≥3 consumptions daily), and a higher peak workload (W) during exercise in both sexes (β women=−0.52 [95% CI, −0.65 to −0.39] and β men=−0.66 [95% CI, −0.80 to −0.53] per SD increase in workload) were associated with lower RWT. Risk factor associations were similar in terms of direction and magnitude when patients with eccentric LVH and cLVH were included in the analysis (Table 4).

**Table 3.**
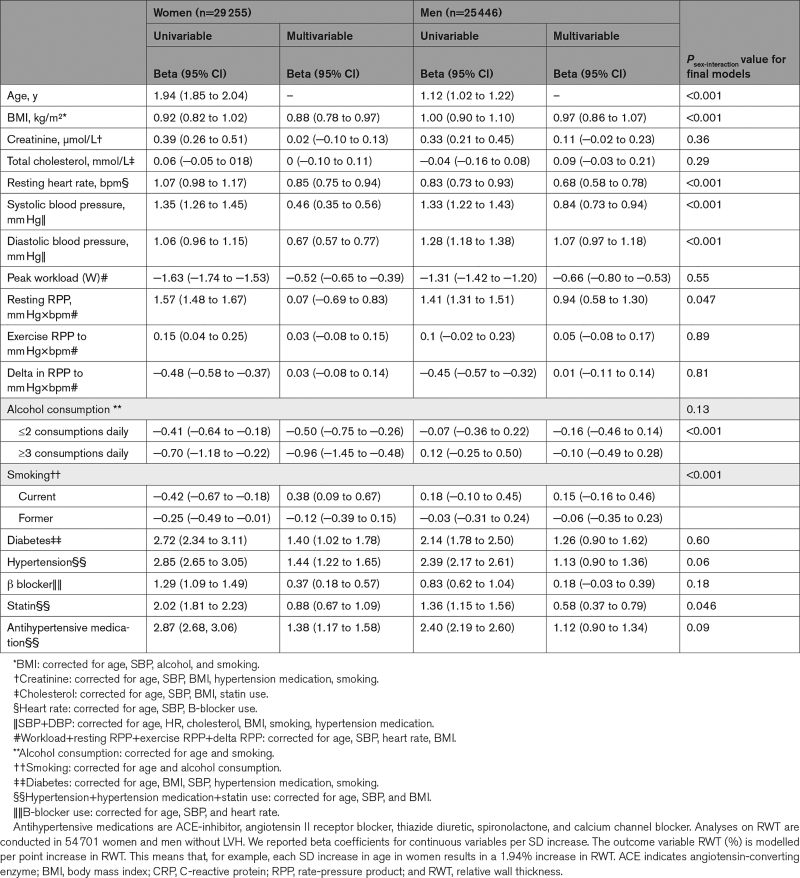
Sex-Stratified Linear Regression Analysis of Risk Factors With RWT (%) in 54 701 Women and Men Without LVH

**Table 4.**
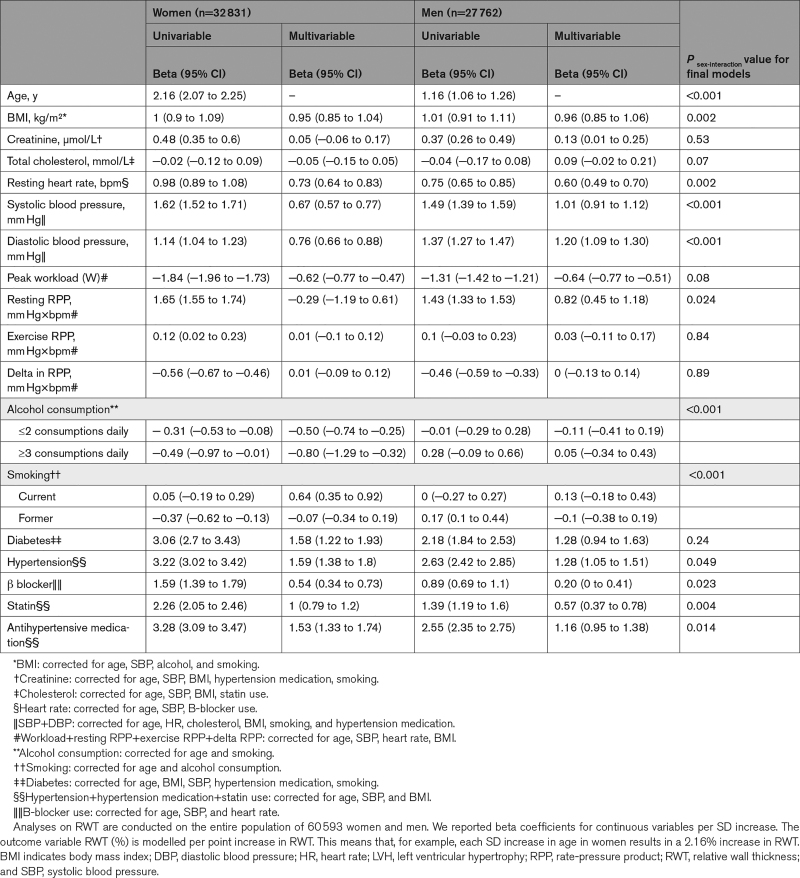
Sex-Stratified Analysis of Risk Factors for RWT (%) in 60 593 Women and Men, Including Individuals With LVH

### Proteomics

**Figure 1.**
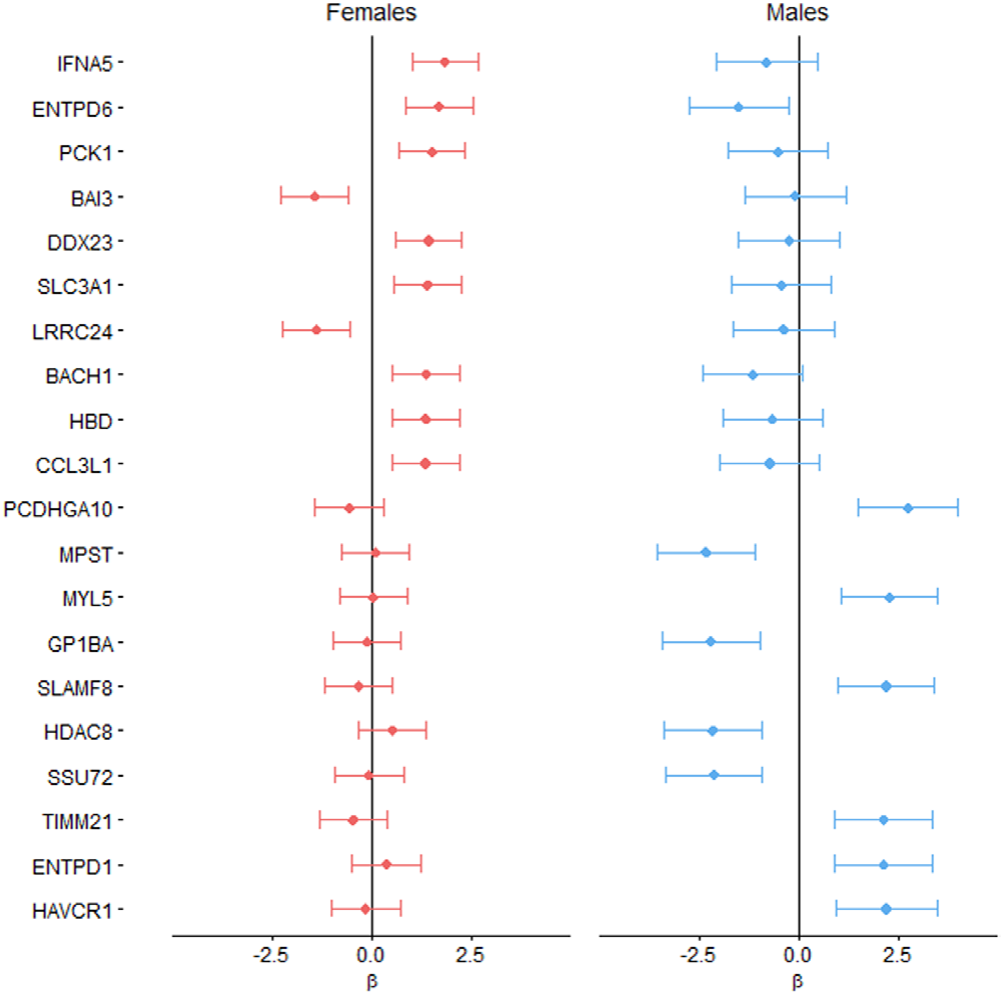
**Women and men comparison of the associations of proteins with relative wall thickness (RWT): associations of top 10 proteins associating with RWT in 520 women and men, respectively.** A negative β represents that a high value of this protein is associated with lower RWT, and a positive β represents that a high value of this protein is associated with higher RWT. Pink bars represent the analysis in women and blue bars in men, and the length of the bars represents the 95% CI of the age-corrected models. Most proteins that associate with a higher RWT in women are neutral or negatively associated in men. Most proteins related to a lower RWT in men are indifferent in women. The * symbol is depicted for proteins that are significantly associated after correction for multiple testing (Benjamini-Hochberg adjusted *P*<0.05). For abbreviations of the proteins, we refer to Table 5.

In the subsample of individuals in whom blood was collected, cRM was present in 44.4% of women and 44.9% of men, and the prevalence of cLVH was 4.2% in women and 4.6% in men (see Tables S5 and S6 for baseline characteristics and risk factor analysis). The group that was also included in the proteomics analysis was not clinically different from the remaining subsample, although small statistical differences were observed (Table S7). In 520 individuals without LVH, the top 20 nominally significantly associated plasma proteins were largely positively associated with RWT in women (17 out of 20). Conversely, in men fewer, 9 of the top 20 proteins, were positively correlated with RWT (Table 5). This was reflected by asymmetry in the volcano plots, significant sex-interaction for most proteins, and no overlap in the top 10 hits between women and men (Figure S4; Table 5; Figure 1). In men, we found that protocadherin gamma-A10 was statistically significantly associated with higher RWT (β=2.72; *P*_adjusted_=0.013) after adjusting for multiple testing, and correcting for age (Table 5). In women, a higher plasma level of IFNA5 (interferon alpha-5) was the top hit (β=1.82; *P*=0.06). After we increased power by addition of women and men with LVH (n=37), the association of IFNA5 reached statistical significance with similar association strength (β=1.94; *P*_adjusted_=0.005; Table 6). In women, each SD increase of normalized IFNA5 levels was associated with a 1.94% increase in RWT. In men, there were no statistically significant findings, and the effect size for protocadherin gamma-A10 decreased (β=2.18; *P*_adjusted_=0.26; Table 6). IFNA5 was not associated with RWT in men (Table 6; Figure S5).

**Table 5.**
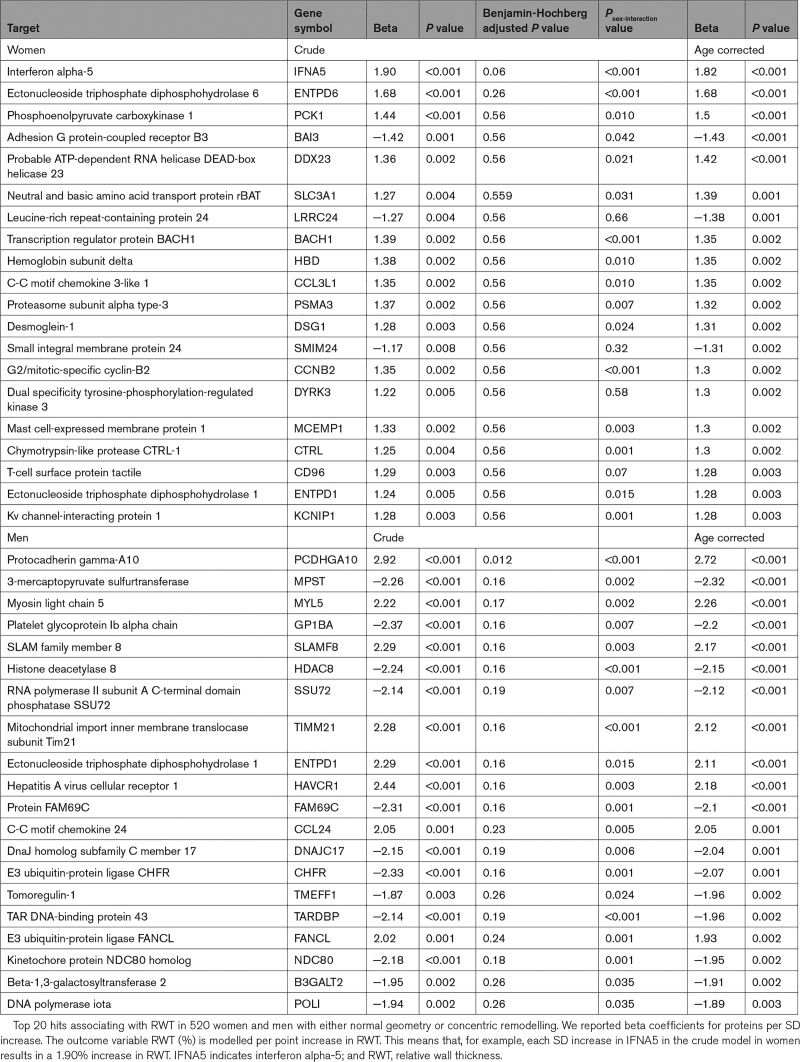
Top 20 Hits Associating 4534 Proteins With RWT in 520 Women and Men With Normal Geometry or Concentric Remodeling in a Sex-Stratified Analysis

**Table 6.**
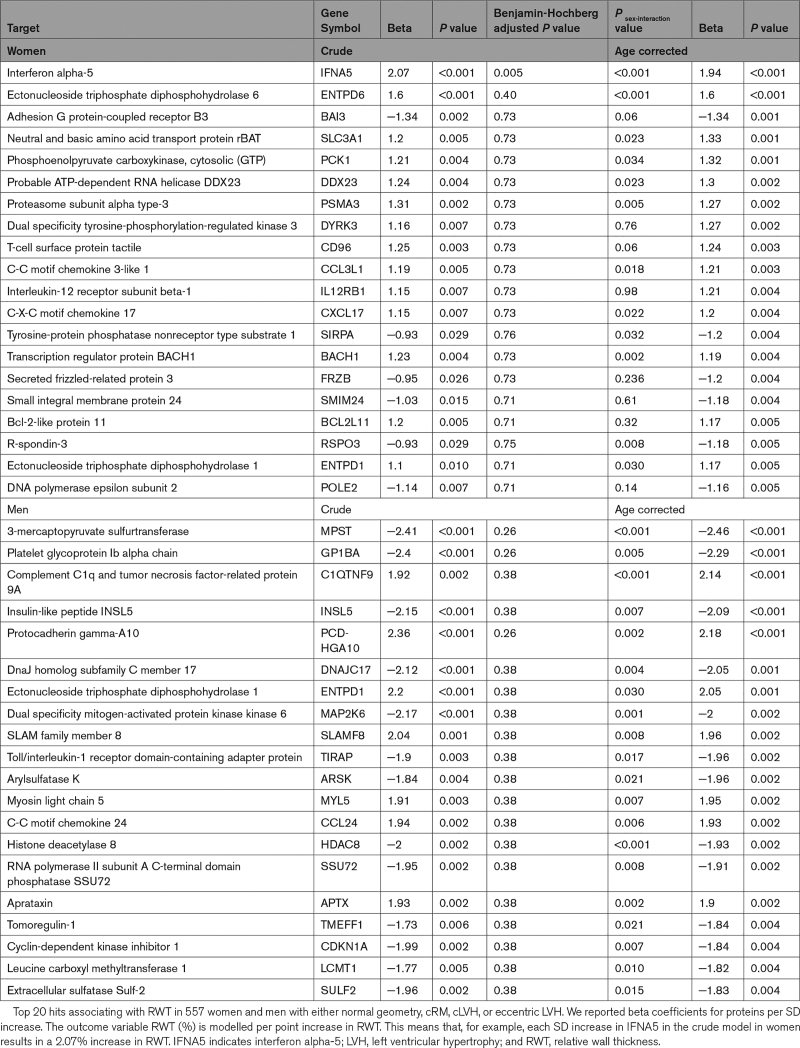
Top 20 Hits in 557 Women and Men in the Sex-Stratified Analysis of 4534 Proteins With RWT, Including Individuals With LVH

**Figure 2.**
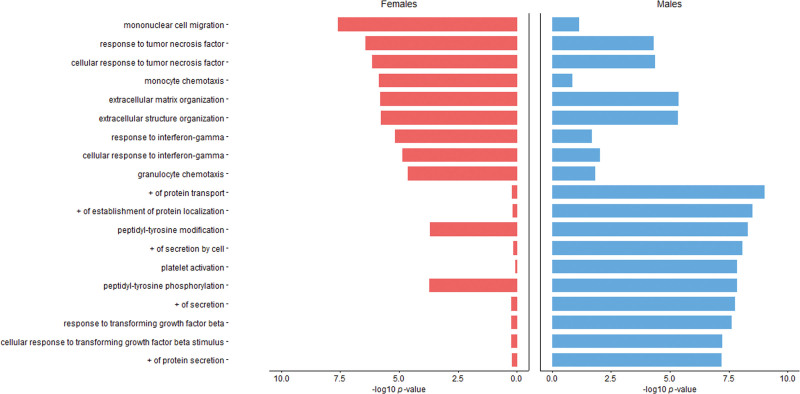
**Women and men comparison of the pathways annotating proteins relating to relative wall thickness (RWT): top 10 pathways annotating proteins that were nominally significantly associated with relative wall thickness for 520 women and men, respectively, are depicted in pink (women) and blue (men).** The strength of the association is represented by the magnitude of the bars as quantified by –log 10 *P* value. + indicates positive regulation.

Pathway analysis revealed that, in women, proteins nominally associated with RWT grouped as mononuclear cell migration (−log 10; *P*=7.59), response to tumor necrosis factor (−log 10; *P*=6.42), monocyte chemotaxis (−log 10; *P*=5.85), extracellular matrix organization (−log 10; *P*=5.79), and interferon-gamma activity (−log 10; *P*=5.18). This is consistent with activation of inflammatory pathways (Figure 2; Figure S6). In men, pathways of protein transport (−log 10; *P*=8.99), protein localization (−log 10; *P*=8.48), and platelet activation (−log 10; *P*=7.82) were found. Comparing the top 10 pathways by sex revealed differences in magnitude of pathway activation associated with RWT (Figure 2).

## DISCUSSION

In a large cohort of individuals at risk of cardiovascular disease, we find a high prevalence of cRM (approximately 1 in 4), which in turn is associated with a higher risk of incident HFpEF and all-cause mortality. Risk factors for a high RWT were similar between women and men but showed statistically significantly stronger associations in women. Yet, activated pathways, annotating proteins relating to RWT, were notably different between sexes. We observed a female predominance of inflammatory pathways marked by an association of interferon alpha-5 with RWT in women (see Figure S1: Central Illustration).

### Incident Heart Failure and Mortality

cRM is commonly conceptualized as a cardiac adaptation to increased afterload caused by conditions such as hypertension and aortic stenosis. The transition of cRM to myocardial failure such as HFpEF is poorly understood but has clinical significance.^3^ We showed that cRM is equally prevalent in both women and men visiting cardiac outpatient clinics, using real-life data. We found that cRM is associated with future development of HFpEF but not associated with overall risk of HF development or HFrEF. Other studies have identified eccentric LVH and cLVH, but not cRM, as markers of risk of incident HF and HFpEF in the general population, but did not report this in a sex-specific manner.^21^ In addition, we also found that cRM is associated with increased all-cause mortality risk. This finding is in contrast to other studies, which did not identify cRM to increase mortality risk,^8,22^ not even in populations with coincident atrial fibrillation,^23^ HFpEF,^5,6^ or prior myocardial infarction.^24^ This discrepancy may be explained by smaller sample size in prior studies, reducing power to detect mildly increased risk. We observed that cRM increased mortality similarly for both sexes. This is in keeping with a magnetic resonance imaging study, which found that cLVH was equally associated with all-cause mortality in women and men.^25^

### Sex Differences in Traditional Risk Factors for Higher RWT

In women, several cardiovascular risk factors had a greater impact on RWT compared with men, including age, heart rate, and hypertension. In women, the magnitude of association between age and RWT was twice as high as in men. During aging, LV mass increases more in women, and cardiomyocytes are better preserved^26^ than in men. This may result in a higher RWT.^27^ Hypertension is an important risk factor for cRM,^27–30^ which we confirm in our study. Women are known to be more susceptible to cRM and diastolic dysfunction as result of pressure overload (eg, aortic stenosis) as compared with men.^10,11^ Consequently, our data are in agreement with the prior observation that relative HF, myocardial infarction, and overall cardiovascular risk attributable to blood pressure is higher in women than in men,^31^ suggesting that sex-specific targets for blood pressure control may be an interesting target to improve cardiovascular prevention in women. Heart rate is slightly higher in women as compared with men to keep up cardiac output given smaller stroke volume.^32^ The stronger association of heart rate with RWT in women was comparable to a study in hypertensive individuals.^33^ Severely reduced stroke volumes due to cRM may drive the attenuated association between increased heart rates and higher RWT in women, highlighting the clinical importance of cRM as target for intervention.

### Plasma Proteomics

Proteomics studies in the field of cardiac remodeling and HFpEF may have importance in understanding of disease biology and identification of therapeutics.^34–40^ Our study adds to prior work as we used a proteomics assay not limited to candidate biomarkers.^41^ We show that RWT is associated with increased circulating proteins involved in mononuclear cell migration, response to tumor necrosis factor, monocyte chemotaxis, extracellular matrix organization, and interferon-gamma activity in women, consistent with activation of inflammatory pathways. Tromp et al^40^ compared biomarker patterns and biological pathways in HFrEF and HFpEF using a cardiovascular protein panel. Inflammatory and extracellular matrix organization pathways were predominantly activated in patients with HFpEF (43% women) compared with patients with HFrEF (26% women), in whom cellular growth and metabolism pathways were upregulated. As HFpEF has a female preponderance, the similarities between studies in inflammatory and immune-related pathway activation suggest a link between onset of cRM and development of HFpEF in women. This supports the idea that biological processes underlying cRM may be sex-dependent.

Two prior proteomic studies focused on sex differences in HFpEF populations.^42,43^ One found proteins involved in extracellular matrix turnover to be differentially expressed between women and men.^42^ The second study showed that proteomic correlates of coronary microvascular dysfunction in patients with HFpEF differed by sex.^43^ Although direct comparison of proteomic studies is complicated, due to protein panel differences and different analysis strategies, accumulating evidence suggests that sex is an important modifier of cardiac remodeling and HFpEF.

We identified higher circulating levels of IFNA5 in women with higher RWT, and this became statistically significant when we added women with LVH to our sample. IFNA5 is a cytokine in the interferon family that plays a role in the immune response to viruses but is also associated with auto-immunity, especially in systemic lupus erythematosus, a condition with a 9:1 female to male prevalence ratio.^44^ TLR7 (toll-like receptor 7), located on the X-chromosome, is one of the pattern recognition receptors responsible for IFN production. Women have 2 X-chromosomes of which one is silenced. This X-chromosome inactivation may be incomplete, resulting in genes that escape X-inactivation. Intriguingly, TLR7 is a gene that frequently escapes X-chromosome inactivation^45^ and may lead to sex-specific increased levels of interferon-α and β.^45^ X-chromosome escape genes have been suggested to explain the high prevalence of autoimmune disease in women as compared with men. Our results inspire the hypothesis that activation of interferon signaling is a result of X-escape mechanisms and may partially explain the increased prevalence of HFpEF in women.

If one considers cRM and cLVH to be early and long-term structural adaptations, respectively, to increased afterload, one could then posit the importance of early intervention in cRM, to prevent deterioration to the higher risk phenotypes of cLVH and HFpEF. Inflammatory biomarkers may have potential for early detection of patients at risk for HFpEF, particularly women. But more importantly, targeting inflammation may provide a window of opportunity for prevention of deterioration toward cLVH or HFpEF. The recent success of SGLT2 (sodium-glucose cotransporter-2) inhibitors to improve prognosis in patients with HFpEF^46^ may hold promise here, since beneficial effects of SGLT2 inhibition include reduced oxidative stress and inflammation, inhibition of cardiac fibrosis, improved endothelial function, and improved filling conditions and diastolic function.^47^ Additionally, statins^48^ and colchicine^49^ are known to target systemic inflammation and are beneficial for prevention of ischemic heart disease, respectively. In the Low Dose Colchicine (LoDoCo) trial subanalysis, however, the effect in women was not convincing, possibly due to small numbers of enrolled women.^49^ Failure to enroll substantial numbers of women in clinical trials continues to hamper understanding of the biologic variability in cardiovascular disease by sex. We communicated in our patient information the need to study women at risk for HFpEF, which resulted in 65.4% inclusion of women in this study, allowing the sex-stratification of our analysis and a deliberate search for sex-specific disease mechanisms.

### Limitations

Despite the large number of plasma proteins assayed, we found only a single protein in women, and no proteins in men, that significantly associated with high RWT in rigorous statistical testing. We acknowledge the limitation that IFNA5 is only statistically significantly associated with a higher RWT in women after adding women with LVH to our analysis. However, the effect size of the association was similar, suggesting a power issue. Our protein pathway analysis findings have not yet been validated, and the prognostic value of IFNA5 for cRM and HFpEF in women needs further investigation.^50^ We are not able to provide reference values for IFNA5 levels, since our data were transformed to be comparable between proteins. Data on infiltrative or restrictive cardiomyopathy were not captured in a standardized manner. Hence, prevalence of these specific disorders was not reported. Finally, our study is limited by incomplete follow-up, which could lead to selection bias. We may have underestimated true heart failure incidence.

### Conclusions

cRM is prevalent in approximately 1 in 4 women and men visiting outpatient cardiology clinics and associated with HFpEF development and mortality risk in both sexes. Known risk factors for cRM were statistically significantly more strongly associated in women than men. Proteomic analysis revealed inflammatory pathway activation in women, with a central role for IFNA5. Differential biologic pathway activation by sex in cRM may contribute to the female predominance of HFpEF and holds promise for identification of new therapeutic avenues for prevention and treatment of HFpEF.

## ARTICLE INFORMATION

### Acknowledgments

All authors have approved the article and agreed with its submission to *Circulation: Heart Failure*.

### Sources of Funding

This study was funded by Dutch Cardiovascular Alliance grant 2020B008 RECONNEXT and 2020B004 IMPRESS.

### Disclosures

None.

### Supplemental Material

Tables S1–S7

Figures S1–S6

## Supplementary Material


