# Supplementary material for: Plasma Proteomic Patterns Show Sex Differences in Early Concentric Left Ventricular Remodeling
Source: Circ Heart Fail. 2023 Jun 29;16(7):e010255. doi: 10.1161/CIRCHEARTFAILURE.122.010255 (PMC10348648; doi:10.1161/CIRCHEARTFAILURE.122.010255)
Supplement: Supplementary file 1 [file hhf-16-e010255-s001.pdf]

SUPPLEMENTAL MATERIAL

Table S1: Percentage of missing values in the total data set and the proteomics subset

Table S2: Unadjusted Cox regression models for the association between of concentric remodeling, eccentric and concentric LVH and HF, HFpEF and mortality

Table S3: Cox regression analyses of concentric remodeling, eccentric and concentric LVH with HFrEF.

Table S4: Logistic Regression analyses for the association between blood pressure and cRM.

Table S5: Baseline characteristics of the proteomics subsample (n= 829) stratified by sex and ventricular geometry class.

Table S6: Sex-stratified linear regression analysis of risk factors with RWT (%) in the proteomics subsample (n= 770).

Table S7: Baseline characteristics of individuals in the subsample that were included in the proteomics analysis and risk factor analyses

Figure S1: Central Illustration

Figure S2: Flowchart for analyses using data from the Cardiology Centers of the Netherlands Outpatient clinics population.

Figure S3: Flowchart for sample selection and quality control in the proteomics subsample

Figure S4: Volcano plots representing proteins negatively and positively associated with relative wall thickness.

Figure S5: Figure S5: The association of Interferon α 5 with higher RWT in women (red) and men (blue).

Figure S6: Pathway analysis of proteins related to relative wall thickness in women and men separately.

Table S1: Percentage of missing values in the total data set and the proteomics subset

	Percentage missingness	
	Proteomics subset (n= 829)	CCN dataset (n= 60,593)
Sex	0	0
Age (years)	0	0
BMI (kg/m²)	0	0
Waist to hip ratio	2.8	NA
Heart rate (bpm)	5.2	0.5
Systolic blood pressure (mmHg)	0.8	1.5
Diastolic blood pressure (mmHg)	0.8	1.4
Maximal workload during exercise testing (Watt)	16.4	24.1
Double product at rest	5.4	2.1
Double product at peak exercise	17	24.6
Delta in double product (peak exercise-rest)	20.9	25.9
Diabetes Mellitus	0	0.9
Hypertension	0	0.8
Hyperlipidemia	0	0.8
Smoking	1	6.8
Alcohol consumption	30.2	9.5
Medication		
β Blocker	0	0
Antihypertensive medication	0	0
Statin	0	0

Laboratory		
Total cholesterol (mmol/L)	10.1	31.4
CRP (mg/L)	27.1	NA
Creatinine (µmol/L)	10.4	30.8
Average amount of missing data	6.7%	8.1%

Abbreviations: BMI, Body Mass Index; CRP, C-reactive protein; NA, not available.

Table S2: Unadjusted Cox regression models for the association between of concentric remodeling, eccentric and concentric LVH and HF, HFpEF and mortality

		Men+ Women		Women	Men
**HF**		HR (95% CI)	p-value sex-interaction	HR (95% CI)	HR (95% CI)
	normal geometry	1	0.012	1	1
	concentric remodelling	1.39 (1.00, 1.94)		1.72 (1.23, 2.40)	1.39 (0.99, 1.94)
	eccentric LVH	4.72 (3.26, 6.85)		2.51 (1.65, 3.80)	4.72 (3.25, 6.86)
	concentric LVH	6.83 (4.77, 9.79		4.16 (2.87, 6.04)	6.85 (4.78, 9.83)
**HFpEF**		HR (95% CI)	p-value sex-interaction	HR (95% CI)	HR (95% CI)
	normal geometry	1	0.031	1	1
	concentric remodelling	2.00 (1.34, 2.98)		1.98 (1.38, 2.83)	1.99 (1.33, 2.97)
	eccentric LVH	3.23 (1.81, 5.75		1.46 (0.82, 2.59)	3.24 (1.82, 5.79)
	concentric LVH	8.47 (5.39, 13.29)		4.05 (2.66, 6.17)	8.50 (5.40, 13.37)
**Mortality**		HR (95% CI)	p-value sex-interaction	HR (95% CI)	HR (95% CI)
	normal geometry	1	<0.001	1	1
	concentric remodelling	1.47 (1.34, 1.62)		1.80 (1.61, 2.00)	1.47 (1.34, 1.62)
	eccentric LVH	3.44 (3.01, 3.93)		3.30 (2.87, 3.79)	3.44 (3.01, 3.93)
	concentric LVH	2.76 (2.37, 3.21)		4.69 (4.13, 5.33)	2.77 (2.38, 3.22)

Abbreviations: HF; heart failure, HFpEF; heart failure with preserved ejection fraction, LVH; left ventricular hypertrophy; HR, hazard ratio; CI, confidence interval

Table S3: Cox regression analyses of concentric remodeling, eccentric and concentric LVH with HFrEF.

		Men+ Women		Women	Men
**HFrEF**	*Crude model*	HR (95% CI)	p-value sex-interaction	HR (95% CI)	HR (95% CI)
	normal geometry	1	0.99	1	1
	concentric remodelling	0.62 (0.32, 1.22)		0.64 (0.21, 1.97)	0.62 (0.32, 1.22)
	eccentric LVH	6.77 (4.10, 11.19)		7.11 (3.52, 14.36)	6.75 (4.08, 11.19)
	concentric LVH	5.14 (2.79, 9.49)		4.87 (2.12, 11.20)	5.16 (2.79, 9.54)
**HFrEF**	*Adjusted model*	HR (95% CI)	p-value sex-interaction	HR (95% CI)	HR (95% CI)
	normal geometry	1	0.79	1	1
	concentric remodelling	0.58 (0.30, 1.14)		0.53 (0.17, 1.66)	0.57 (0.29, 1.13)
	eccentric LVH	5.62 (3.37, 9.36)		6.03 (2.88, 12.62)	5.51 (3.29, 9.25)
	concentric LVH	4.63 (2.48, 8.63)		3.57 (1.47, 8.69)	4.56 (2.43, 8.58)

Abbreviations: HFrEF, heart failure with reduced ejection fraction; LVH, left ventricular hypertrophy; HR, hazard ratio; CI, confidence interval.

Corrected for: age, systolic blood pressure, body mass index, diabetes, smoking status and kidney function

Table S4: Logistic Regression analyses for the association between blood pressure and cRM.

	Women (n= 29,255) OR (95% CI)	Men (n= 25,446) OR (95% CI)	p-value sex-interaction
Hypertension*	1.06 (1.05, 1.08)	1.05 (1.04, 1.07)	0.29
SBP†	1.02 (1.02, 1.03)	1.04 (1.03, 1.04)	<0.001
DBP†	1.03 (1.02, 1.03)	1.05 (1.04, 1.05)	<0.001

Abbreviations: SBP, systolic blood pressure; DBP, diastolic blood pressure; OR, odds ratio; CI, confidence interval. SBP and DBP are modelled per standard deviation increase. * Corrected for age, SBP and body mass index. † Corrected for age, heart rate, cholesterol, body mass index, smoking, hypertension medication

Table S5: Baseline characteristics of the proteomics subsample (n= 829) stratified by sex and ventricular geometry class.

	normal geometry n= 399 (48.1%)			concentric remodelling n= 371 (44.7%)			eccentric LVH n= 23 (2.8%)			concentric LVH n= 36 (4.3%)		
	men	women	p-value	men	women	p-value	men	women	p-value	men	women	p-value
n (%) by sex	126 (48.3)	273 (48.1)		116 (44.4)	255 (44.9)		7 (2.7)	16 (2.8)		12 (4.6)	24 (4.2)	
Age (years)	62 (10)	61 (9)	0.57	63 (10)	64 (8)	0.67	70 (6)	65 (11)	0.31	67 (11)	69 (10)	0.61
BMI (kg/m²)	27.0 (3.4)	26.8 (4.6)	0.81	27.3 (3.7)	27.1 (4.7)	0.62	25.9 (3.7)	27.7 (4.0)	0.34	27.5 (2.4)	28.5 (5.5)	0.58
Waist to hip ratio	0.96 (0.07)	0.89 (0.07)	<0.001	0.97 (0.07	0.89 (0.07)	<0.001	0.98 (0.07)	0.91 (0.07)	0.039	0.96 (0.06)	0.96 (0.10)	0.93
Heart rate (bpm)	70 (12)	71 (11)	0.55	73 (13)	74 (11)	0.55	66 (14)	70 (13)	0.49	71 (17)	69 (14)	0.73
Systolic blood pressure (mmHg)	148 (18)	142 (19)	0.002	150 (20)	147 (20)	0.30	156 (16)	141 (17)	0.08	157 (25)	163 (21)	0.45
Diastolic blood pressure (mmHg)	88 (10)	84 (10)	<0.001	90 (11)	87 (11)	0.008	86 (10)	85 (11)	0.83	91 (12)	90 (11)	0.79
Peak workload exercise (W)	188 (45)	128 (34)	<0.001	179 (47)	122 (33)	<0.001	170 (49)	121 (51)	0.09	151 (68)	114 (40)	0.12
RPP at rest	10352 (2156)	10066 (2131)	0.22	10916 (2486)	10953 (2314)	0.90	10300 (2164)	9902 (1998)	0.69	11258 (3782)	11253 (2892)	0.99
RPP at peak exercise	30632 (7015)	26990 (6478)	<0.001	31479 (6571)	26399 (6090)	<0.001	29320 (9102)	25176 (6947)	0.32	26700 (8785)	26949 (9109)	0.95
Delta RPP (peak-rest)	20588 (7002)	17124 (6291)	<0.001	20690 (6667)	15684 (5761)	<0.001	15353 (1870)	15574 (7762)	0.96	17193 (9471)	15320 (8815)	0.66
Diabetes Mellitus (n (%))	6 (4.8)	14 (5.1)	1	14 (12.1)	20 (7.8)	0.27	2 (28.6)	2 (12.5)	0.56	1 (8.3)	1 (4.2)	1
Hypertension (n (%))	60 (47.6)	131 (48.0)	1	75 (64.7)	162 (63.5)	0.93	5 (71.4)	10 (62.5)	1	8 (66.7)	21 (87.5)	0.19
Hyperlipidemia (n (%))	47 (37.3)	100 (36.6)	0.99	41 (35.3)	118 (46.3)	0.06	5 (71.4)	7 (43.8)	0.37	5 (41.7)	13 (54.2)	0.73
Smoking (n (%))			0.45			0.60			0.18			0.06
never	49 (38.9)	120 (44.3)		36 (32.1)	95 (37.5)		1 (14.3)	8 (50.0)		3 (25.0)	15 (62.5)	
current	13 (10.3)	20 (7.4)		11 (9.8)	25 (9.9)		0 (0.0)	0 (0.0)		4 (33.3)	2 (8.3)	
former	64 (50.8)	131 (48.3)		65 (58.0)	133 (52.6)		6 (85.7)	8 (50.0)		5 (41.7)	7 (29.2)	
Alcohol consumption (n (%))			0.008			<0.001			0.76			0.60
never	7 (7.4)	39 (21.2)		4 (4.9)	36 (20.2)		2 (28.6)	3 (30.0)		2 (22.2)	4 (25.0)	

≤ 2 consumptions daily	69 (73.4)	123 (66.8)		62 (76.5)	136 (76.4)		4 (57.1)	7 (70.0)		5 (55.6)	11 (68.8)	
≥ 3 consumptions daily	18 (19.1)	22 (12.0)		15 (18.5)	6 (3.4)		1 (14.3)	0 (0.0)		2 (22.2)	1 (6.2)	
Echocardiography												
IVSD at end-diastole (mm)	9.4 (1.3)	8.5 (1.2)	<0.001	10.7 (1.5)	10.0 (1.3)	<0.001	11.6 (2.0)	10.4 (1.0)	0.06	14.8 (2.9)	12.0 (1.5)	<0.001
LVD at end-diastole (mm)	49 (5)	45 (4)	<0.001	44 (5)	41 (4)	<0.001	57 (5)	52 (5)	0.021	50 (4)	46 (4)	0.042
LVPWD at end-diastole (mm)	8.9 (1.1)	8.2 (0.9)	<0.001	10.8 (1.2)	10.0 (1.0)	<0.001	10.6 (0.5)	9.6 (0.7)	0.005	13.4 (1.5)	11.5 (0.8)	<0.001
Left ventricular ejection fraction (%)	68 (8)	68 (7)	0.72	66 (8)	67 (8)	0.11	65 (16)	66 (11)	0.86	67 (11)	67 (9)	0.90
Average E/e' ratio	8.4 (2.3)	9.2 (2.5)	0.002	8.6 (2.0)	9.7 (2.6)	<0.001	9.0 (2.0)	11.2 (2.7)	0.061	13.0 (6.8)	11.5 (3.3)	0.36
Left atrial volume index (ml/m2)	26 (9)	25 (8)	0.78	25 (9)	24 (9)	0.48	30 (12)	28 (12)	0.76	29 (9)	35 (26)	0.43
Left ventricular mass index (g/m²)	76 (16)	67 (12)	<0.001	80 (17)	72 (14)	<0.001	132 (17)	104 (7)	<0.001	137 (20)	109 (14)	<0.001
Relative wall thickness (%)	36 (4)	36 (4)	0.56	50 (8)	49 (6)	0.45	37 (3)	37 (4)	0.87	55 (9)	50 (6)	0.06
Medication												
β Blocker (n (%))	16 (12.7)	43 (15.8)	0.52	7 (6.0)	39 (15.3)	0.019	2 (28.6)	3 (18.8)	0.62	1 (8.3)	7 (29.2)	0.22
Antihypertensive medication (n (%))	36 (28.6)	64 (23.4)	0.33	43 (37.1)	99 (38.8)	0.84	4 (57.1)	7 (43.8)	0.67	6 (50.0)	15 (62.5)	0.50
Statin (n (%))	27 (21.4)	43 (15.8)	0.21	29 (25.0)	58 (22.7)	0.73	3 (42.9)	2 (12.5)	0.14	4 (33.3)	4 (16.7)	0.40
Laboratory												
Total cholesterol (mmol/L)	5 (1)	5 (1)	0.71	5 (1)	5 (1)	<0.001	5 (1)	6 (1)	0.45	5 (1)	5 (1)	0.91
CRP (mg/L)	1.4 [0.7, 2.4]	1.5 [0.7, 3.3]	0.24	1.3 [0.7, 2.9]	1.4 [0.7, 3.6]	0.67	1.6 [1.2, 3.5]	1.6 [1.3, 4.8]	0.62	1.2 [1.0, 4.0]	1.4 [0.9, 2.7]	0.64
Creatinine (µmol/L)	81 (14)	64 (11)	<0.001	78 (13)	65 (10)	<0.001	93 (22)	63 (6)	<0.001	77 (14)	67 (16)	0.07

Abbreviations: BMI, Body Mass Index; RPP, Rate-pressure product; IVSD, Interventricular septal diameter; LVD, Left ventricular internal dimension; LVPWD, left ventricular posterior wall diameter; CRP, C-reactive protein.

Antihypertensive medication are ACE-inhibitor, angiotensin II receptor blocker, thiazide diuretic, spironolactone and calcium channel blocker

The table represents 829 individuals that had no missing data on concentric remodeling.

P-values are calculated comparing women and men within each geometry class. Parametric and non-parametric tests are used for continuous variables based on normality of the distribution. For counts the Chi Square or Fisher exact test was used.

Table S6: Sex-stratified linear regression analysis of risk factors with RWT (%) in the proteomics subsample (n= 770).

	Women (n= 528)		Men (n= 242)		
	univariable	multivariable	univariable	multivariable	p-value sex interaction final model
	Beta (95% CI)	Beta (95% CI)	Beta (95% CI)	Beta (95% CI)	
Age (years)	**1.17 (0.00, 2.33)**	-	1.59 (-0.91, 2.27)	-	0.38
BMI (kg/m²) *	0.74 (-0.43, 1.91)	1.01 (-0.21, 2.23)	0.06 (-0.63, 0.75)	-0.06 (-0.78, 0.65)	0.10
WHR *	1.28 (-0.06, 2.63)	1.21 (-0.17, 2.59)	-0.33 (-0.75, 0.68)	-0.46 (-1.17, 0.26)	0.026
Creatinine (µmol/L) †	0.38 (-0.80, 1.57)	0.20 (-0.99, 1.39)	0.68 (-0.04, 1.41)	0.40 (-0.32, 1.12)	0.67
Total cholesterol (mmol/L) ‡	-1.04 (-2.21, 0.13)	-0.84 (-2.14, 0.47)	0.31 (-0.40, 1.41)	0.26 (-0.48, 0.99)	0.13
log(CRP) ‡	0.83 (-0.58, 2.23)	0.58 (-0.85, 2.02)	0.24 (-0.55, 1.04)	0.16 (-0.71, 1.03)	0.60
Resting heart rate (bpm) §	2.19 (0.96, 3.42)	**1.93 (0.69, 3.17)**	1.16 (0.47, 1.85)	**0.93 (0.24, 1.63)**	0.23
Systolic blood pressure (mmHg) ‖	1.32 (0.16, 2.49)	0.79 (-0.43, 2.02)	1.43 (0.75, 2.12)	0.71 (-0.02, 1.44)	0.87
Diastolic blood pressure (mmHg) ‖	1.59 (0.43, 2.75)	**1.25 (0.08, 2.41)**	1.29 (0.61, 1.98)	**0.97 (0.26, 1.67)**	0.66
Peak workload (W) #	-0.70 (-1.96, -0.56)	0.15 (-1.37, 1.68)	-1.06 (-1.78, -0.33)	-0.01 (-0.88, 0.85)	0.86
Resting RPP (mmHg*bpm) #	2.51 (1.30, 3.71)	9.97 (-1.90, 21.85)	1.71 (1.03, 2.39)	3.42 (-3.53, 10.37)	0.37

Exercise RPP (mmHg*bpm) #	0.96 (-0.32, 2.24)	0.86 (-0.48, 2.20)	0.11 (-0.63, 0.86)	-0.02 (-0.85, 0.81)	0.26
Delta in RPP (mmHg*bpm) #	0.02 (-1.27, 1.30	0.82 (-0.53, 2.18)	-0.53(-1.28, 0.22)	-0.03 (-0.83, 0.77)	0.27
Alcohol consumption **					0.44
≤ 2 consumptions daily	1.02 (-5.15, 7.19)	0.49 (-5.88, 6.86)	-0.05 (-1.93, 1.84)	-0.51 (-2.42, 1.40)	
≥ 3 consumptions daily	1.06 (-5.70, 7.82)	0.41 (-6.64, 7.46)	-2.56 (-5.81, 0.69)	**-3.44 (-6.71, -0.16)**	
Smoking ††					0.44
former	-0.36 (-2.93, 2.22)	-1.00 (-3.72, 1.73)	0.90 (-0.56, 2.36)	1.31 (-0.18, 2.80)	
current	-2.24 (-6.53, 2.05)	-2.48 (-6.84, 1.88)	-0.01 (-2.61, 2.59)	0.76 (-1.82, 3.34)	
Diabetes Mellitus ‡‡	3.08 (-1.17, 7.32)	2.82 (-1.47 7.12)	0.92 (-1.9, 3.73)	-0.72 (-3.57, 2.13)	0.15
Hypertension §§	3.23 (0.90, 5.55)	2.30 (-0.21, 4.80)	3.16 (1.79, 4.52)	**2.30 (0.77, 3.84)**	0.99
β blocker ‖‖	-3.89 (-7.86, 0.08)	-3.08 (-7.00, 0.84)	-0.74 (-2.65, 1.17)	-0.75 (-2.64, 1.13)	0.27
Statin §§	1.49 (-1.29, 4.26)	0.61 (-2.24, 3.47)	2.01(0.26, 3.76)	1.20 (-0.56, 2.95)	0.72
Antihypertensive medications §§	1.62 (-0.87, 4.12)	0.70 (-1.87, 3.26)	3.67 (2.20, 5.13)	**2.83 (1.32, 4.34)**	0.99

Abbreviations: RWT, relative wall thickness; BMI, Body Mass Index; CRP, C-reactive protein; bpm, beats per minute; RPP, Rate-pressure product; CI.

Confidence interval.

Antihypertensive medications are ACE-inhibitor, angiotensin II receptor blocker, thiazide diuretic, spironolactone and calcium channel blocker. Bold values represent significant findings from the final models. Analyses on RWT are conducted in 770 women and men without LVH. We reported beta coefficients for continuous variables per standard deviation increase. The outcome variable RWT (%) is modelled per point increase in RWT. This means that, for example, each SD increase in age in women results in a 1.17% increase in RWT.

* BMI+WHR: corrected for age, SBP, alcohol and smoking, † creatinine: corrected for age, SBP, BMI, hypertension medication, smoking, ‡ CRP + cholesterol: corrected for age, SBP, BMI, statin use, § Heart rate: corrected for age, SBP, B-blocker use, ‖ SBP + DBP: corrected for age, HR, cholesterol, BMI, smoking, hypertension medication, ¶ workload + resting rpp + exercise rpp + delta rpp: corrected for age, SBP, heart rate, BMI, ** alcohol consumption: corrected for age and smoking, †† smoking: corrected for age and alcohol consumption, ‡‡ Diabetes: corrected for age, BMI, SBP, hypertension medication, smoking, §§ Hypertension + hypertension medication + statin use: corrected for age, SBP and BMI, ‖‖B-blocker use: corrected for age, SBP and heart rate

Supplemental Table 7: Baseline characteristics of individuals in the subsample that were included in the proteomics analysis and risk factor analysis.

n	Included in proteomics analysis and risk factor analysis<br>n= 557	Included in risk factor analysis only<br>n= 272	p-value
Women (n (%))	364 (65.4)	204 (75.0)	0.006
Age (years)	63 (9)	63 (9)	0.61
BMI (kg/m²)	27.1 (4.5)	27.1 (4.3)	0.94
Waist to hip ratio	0.91 (0.07)	0.92 (0.08)	0.007
Heart rate (bpm)	72 (12)	72 (12)	0.33
Systolic blood pressure (mmHg)	147 (20)	145 (20)	0.08
Diastolic blood pressure (mmHg)	87 (10)	86 (11)	0.28
Maximal workload during exercise testing (Watt)	141 (45)	143 (49)	0.58
Double product at rest	10553 (2303)	10533 (2368)	0.91
Double product at peak exercise	28236 (6554)	27292 (7311)	0.10
Delta in double prouct (peak exercise-rest)	17882 (6445)	16955 (7068)	0.09
Diabetes Mellitus (n (%))	42 (7.5)	18 (6.6)	0.74
Hypertension (n (%))	314 (56.4)	158 (58.1)	0.69
Hyperlipidemia (n (%))	223 (40.0)	113 (41.5)	0.73
Smoking (n (%))			0.035
never	203 (36.9)	124 (45.8)	
current	56 (10.2)	19 (7.0)	
former	291 (52.9)	128 (47.2)	
Alcohol consumption (%)			0.13
never	56 (14.7)	41 (20.6)	

≤2 consumptions daily	277 (72.9)	140 (70.4)	
>2 consumptions daily	47 (12.4)	18 (9.0)	
Echocardiography			
IVSD at end-diastole (mm)	9.7 (1.7)	9.6 (1.8)	0.73
LVD at end-diastole (mm)	45 (5)	44 (5)	0.017
LVPWD at end-diastole (mm)	9.4 (1.5)	9.4 (1.6)	0.96
Left ventricular ejection fraction (%)	67 (8)	67 (7)	0.81
average E/e' ratio	9.1 (2.7)	9.4 (2.7)	0.12
Left atrial volume index (ml/m2)	26 (9)	25 (11)	0.44
Left ventricular mass index (g/m²)	76 (19)	74 (18)	0.25
RWT (%)	43 (8)	43 (9)	0.15
Remodeling (%)			0.74
Normal geometry	275 (49.4)	124 (45.6)	
Concentric remodeling	242 (43.4)	129 (47.4)	
Eccentric LVH	16 (2.9)	7 (2.6)	
Concentric LVH	24 (4.3)	12 (4.4)	
Medication			
β Blocker (n (%))	79 (14.2)	39 (14.3)	1
Antihypertensive medication (n (%))	184 (33.0)	90 (33.1)	1
Statin (n (%))	124 (22.3)	46 (16.9)	0.09
Laboratory			
Total cholesterol (mmol/L)	5 (1)	5 (1)	0.40
CRP (mg/L)	2.0 [0.9, 5.5]	1.3 [0.7, 2.8]	0.002
Creatinine (µmol/L)	69 (14)	70 (14)	0.59

Abbreviations: BMI, Body Mass Index; CRP, C-reactive protein; IVSD, Interventricular septal diameter; LVD, Left ventricular internal dimension; LVPWD, left ventricular posterior wall diameter; RPP, Rate-pressure product.

Antihypertensive medication are ACE-inhibitor, angiotensin II receptor blocker, thiazide diuretic, spironolacton and calcium channel blocker

The table represents 829 individuals that had no missing data on concentric remodeling

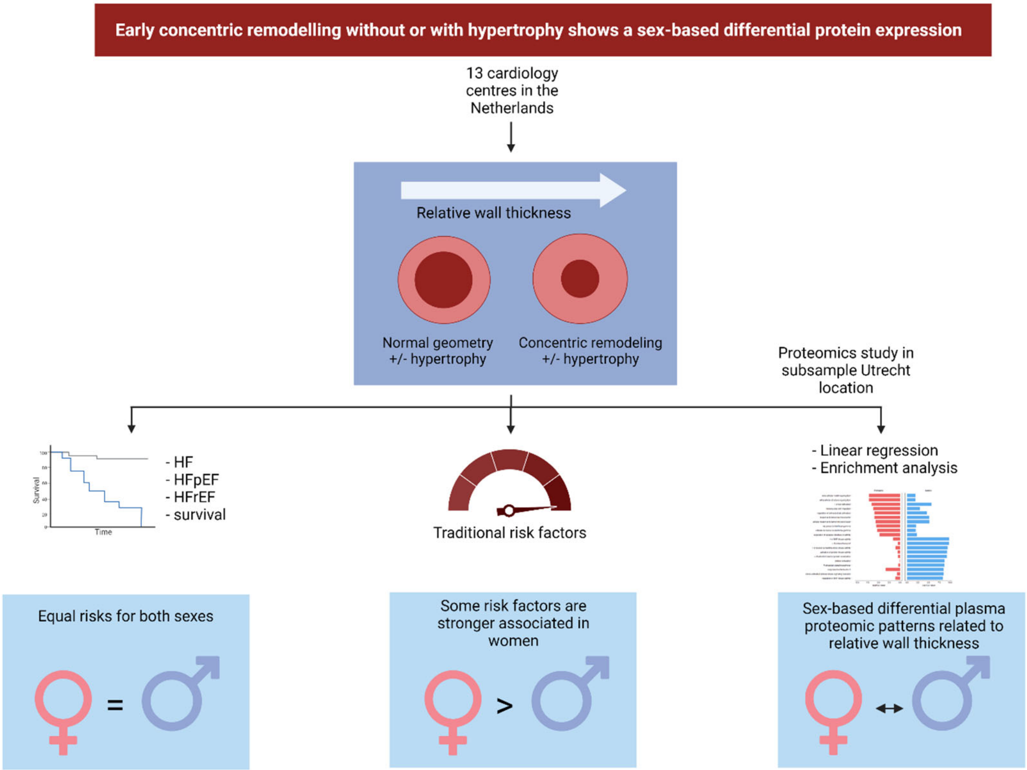

Figure S1: Central Illustration

Concentric remodeling confers similar risks between women and men for development of heart failure with preserved ejection fraction (HFpEF) and increases risk of death similarly in both sexes. Contributing risk factors for increased relative wall thickness (RWT) differ statistically significantly in strength of association by sex. Plasma proteomic analysis shows differences in circulating proteins by sex. Several of the top 20 proteins associated with higher relative wall thickness in women are associated with lower relative wall thickness in men. Higher circulating levels of interferon alpha-5 (IFNA5) are associated with higher RWT in women only.

Figure S2: Flowchart for analyses using data from the Cardiology Centers of the Netherlands Outpatient clinics population.

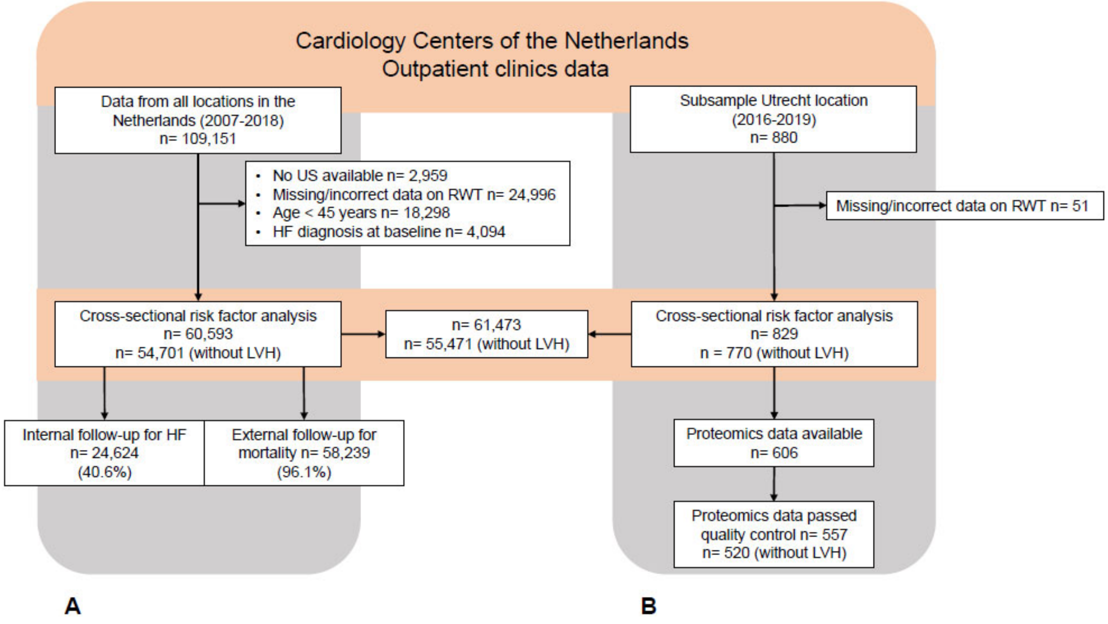

Description of selection of participants for heart failure, survival and proteomics analysis. Panel A: Participants included in HF and survival analysis. Panel B: Participants included in proteomics analysis. Cross-sectional risk factors analysis for relative wall thickness was performed in both cohorts. Abbreviations: HF, heart failure; US, ultrasound.

Figure S3: Flowchart for sample selection and quality control in the proteomics subsample

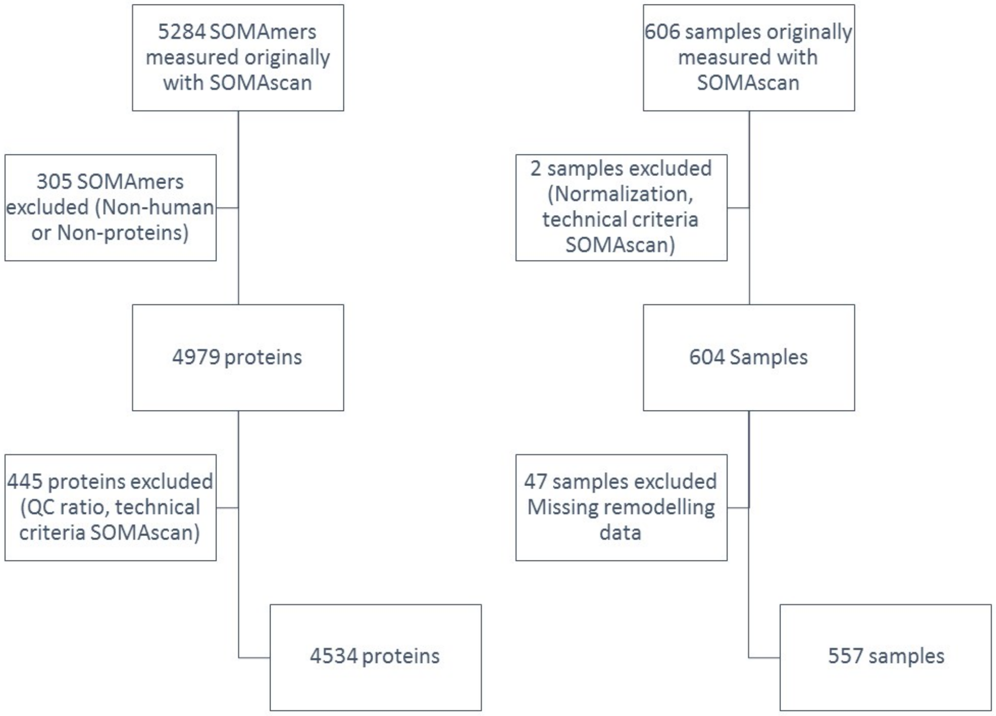

Flowchart describing protein and sample selection respectively. QC; quality control

Figure S4: Volcano plots representing proteins negatively and positively associated with relative wall thickness.

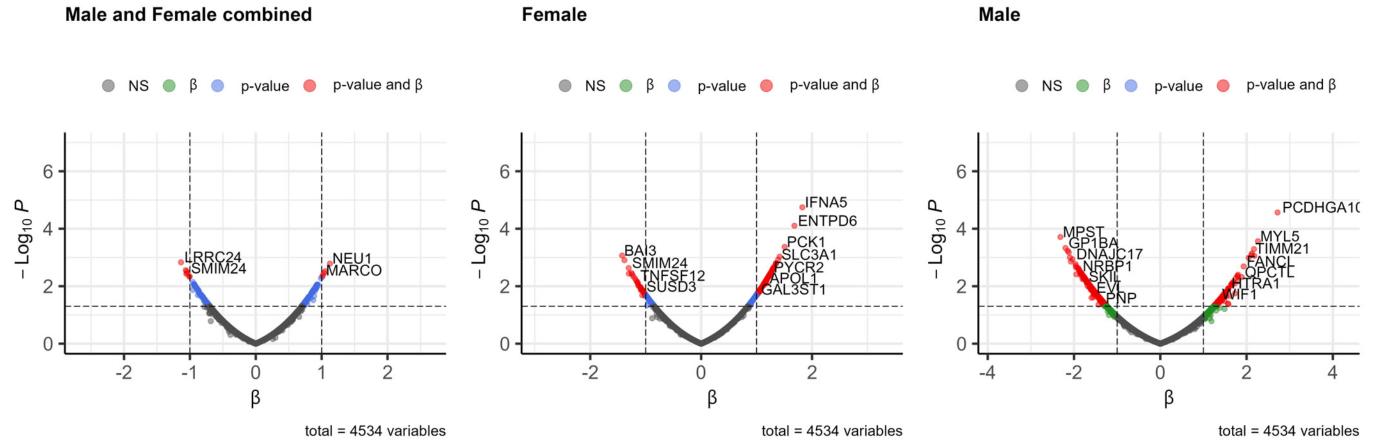

Volcano plots with on the x-axis displaying the direction of the association of the protein with relative wall thickness. A negative β represents that a high value of this protein is associated with lower relative wall thickness, and a positive β represents that a high value of this protein is associated with higher relative wall thickness. On the y-axis the significance of the association is shown. When separating the plot for males and females there remains asymmetry in the female plot, meaning that in females more proteins are associated with higher relative wall thickness.

Figure S5: The association of Interferon α 5 with higher RWT in women (red) and men (blue).

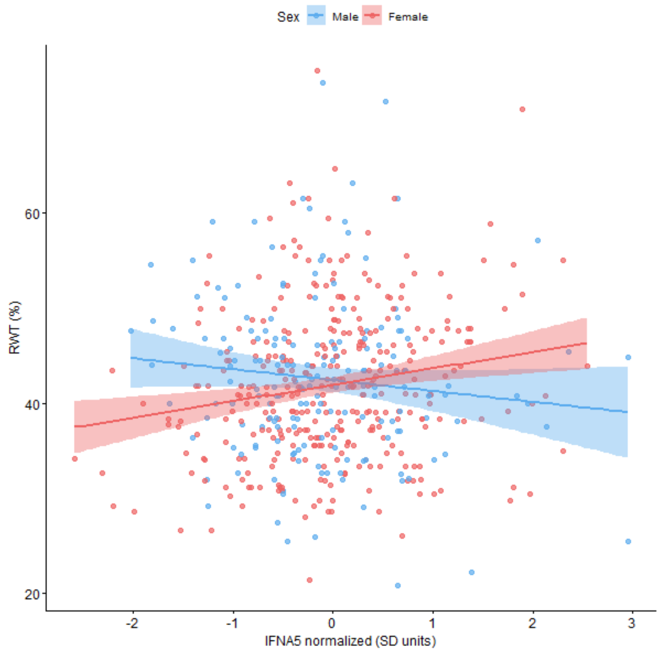

An opposite directed association of IFNA5 with relative wall thickness for women and men is found ($p_{sex\text{-}interaction}$ <0.001). Abbreviations: IFNA5, Interferon α 5; RWT, relative wall thickness; SD, standard deviation.

Figure S6: Pathway analysis of proteins related to relative wall thickness in women and men separately.

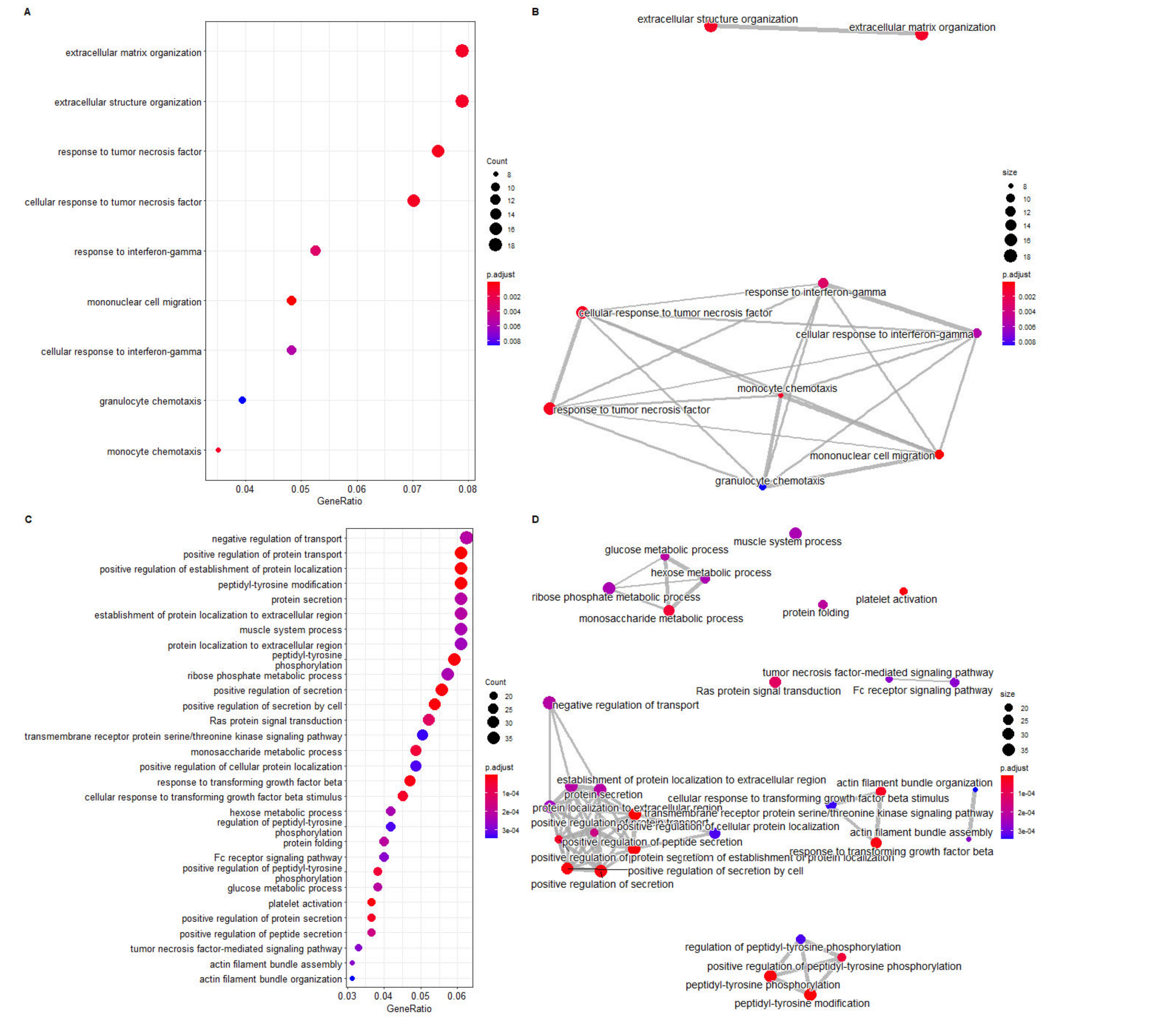

A and B: Pathway analysis in females, C and D: Pathway analysis in males. Pathway analysis was performed for females and males separately, using proteins that associated with RWT. In females there was a high expression of proteins involved in processes related to cell adhesion, extracellular matrix organization and tumor necrosis factor and interferon-gamma activity. Proteins influencing the process of intracellular protein localization and kinase activity were most frequently expressed in males, also MAP kinase and the IL1/Fc-response clusters were only active in males. Abbreviations: MAP, mitogen activated protein; RWT, relative wall thickness.
